# Early versus Late Pars Plana Vitrectomy in Vitreous Hemorrhage: A Systematic Review

**DOI:** 10.3390/jcm12206652

**Published:** 2023-10-20

**Authors:** Filippo Confalonieri, Gianmaria Barone, Vanessa Ferraro, Giacomo Ambrosini, Alessandro Gaeta, Beáta Éva Petrovski, Xhevat Lumi, Goran Petrovski, Alessandra Di Maria

**Affiliations:** 1 Department of Ophthalmology, IRCCS Humanitas Research Hospital, 20089 Milan, Italy; gianmaria.barone@humanitas.it (G.B.); vanessa.ferraro@humanitas.it (V.F.); alessandra.di_maria@humanitas.it (A.D.M.); 2 Department of Biomedical Sciences, Humanitas University, 20090 Milan, Italy; 3 Center for Eye Research and Innovative Diagnostics, Department of Ophthalmology, Institute for Clinical Medicine, University of Oslo, Kirkeveien 166, 0450 Oslo, Norway; beata.petrovski@medisin.uio.no (B.É.P.); goran.petrovski@medisin.uio.no (G.P.); 4 Department of Ophthalmology, Oslo University Hospital, Kirkeveien 166, 0450 Oslo, Norway; 5 Ophthalmology Unit, Department of Experimental Medicine, University of Rome “Tor Vergata”, 00133 Rome, Italy; giacomo.ambrosini@ptvonline.it; 6 Department of Internal Medicine and Medical Specialties (DIMI), Università di Genova, Viale Benedetto XV, 16132 Genova, Italy; s5102725@studenti.unige.it; 7 Eye Hospital, University Medical Centre Ljubljana, Zaloška Cesta 2, 1000 Ljubljana, Slovenia; xhevat.lumi@kclj.si; 8 Department of Ophthalmology, University of Split School of Medicine and University Hospital Centre, 21000 Split, Croatia

**Keywords:** vitreoretinal surgery, pars plana vitrectomy, vitreous hemorrhage, proliferative diabetic retinopathy, eye surgery

## Abstract

**Background**: Vitreous hemorrhage (VH) is a common vitreoretinal condition causing impairment of vision due to various etiologies. No consensus exists on the best timing for performing pars plana vitrectomy (PPV) in fundus-obscuring VH. **Materials and Methods**: Following the Preferred Reporting Items for Systematic Reviews and Meta-Analyses (PRISMA) standards, we conducted a systematic review of the timing of PPV in VH. We assessed the strength of the evidence using the Grading of Recommendations Assessment, Development, and Evaluation (GRADE) approach for all the included publications, in accordance with the 2011 Oxford Centre for Evidence-Based Medicine (OCEBM) recommendations. **Results**: A total of 1731 articles were identified. Following the removal of duplicates and screening of abstracts, 1203 articles remained. Subsequently, a comprehensive full-text review of 30 articles was conducted. Ultimately, 18 articles met the predefined inclusion criteria. **Conclusions**: Despite the small number of studies on the timing of treatment for VH, the advantage of early over late PPV seems to be a reasonable approach in selected cases, and it might be considered modern standard care.

## 1. Introduction

Vitreous hemorrhage (VH), also known as vitreous bleeding, is a medical condition that occurs when blood leaks into the vitreous humor [1]. It can have various causes, the most frequent of which are posterior vitreous detachment (PVD) with or without retinal tear, rhegmatogenous retinal detachment, diabetic retinopathy, and ocular trauma. Other causes that should not be underestimated include retinal vein occlusion, sickle cell retinopathy, retinal vasculitis, Terson syndrome (TS), Valsalva retinopathy, and rarer pathological conditions such as retinopathy of prematurity (ROP), familial exudative vitreoretinopathy (FEVR), and blood dyscrasias [2]. The symptomatology of VH varies upon its severity: mild occurrence can manifest with symptoms of floaters, photopsia, and blurred vision, while more severe hemorrhages, such as in fundus-obscuring cases, can cause a substantial decrease in visual acuity and loss of vision. Generally, the latter described conditions are caused more often by retinal tears or proliferative diabetic retinopathy (PDR) [3].

Determining the optimal timing for surgical intervention in fundus-obscuring VH remains an ongoing challenge. Even though several specialists prefer a conservative treatment, it has been shown that VH carries a potential risk of turning into a retinal detachment (RD), glaucoma, or pigmentary retinopathy [4,5,6]. In particular, it is known that delayed surgery carries the risk of multiple complications, such as retinal tears, epiretinal membrane formation, significant intraoperative bleeding, and recurrent VHs. Additionally, lens opacification is typical, with an increased likelihood of requiring cataract extraction surgery [7]. To avoid these complications, many clinicians prefer to anticipate surgical intervention with early pars plana vitrectomy (PPV) [8].

In the setting of a severe fundus-obscuring VH, clinical examination is made difficult by the inability to examine the fundus. In these cases, it is necessary to perform B-scan ultrasonography to determine or exclude the presence of retinal tears and RD [9]. Given the high risk of underlying retinal pathologies, it is essential to consider PPV as a first-line intervention rather than simply monitoring the patient. Decision making relies on striking a balance between surgical risk and the repercussions of postponed detection of retinal tears [10].

In recent years, there has been a shift towards early vitrectomy, as innovative tools and technical improvements have significantly reduced the risk of intra- and post-operative complications [6,11]. Vitrectomy not only allows for the removal of vitreous opacities but also the removal of vitreous scaffolds, improving both visual acuity (VA) and reducing the risk of complications related to vitreous hemorrhage [12].

In this systematic review, we analyzed the literature to evaluate the difference between early vitrectomy and delayed/late vitrectomy in patients with dense VH. Our goal was to obtain an evidence-based conclusion on the best treatment approach for patients with dense VH.

## 2. Materials and Methods

A systematic review was performed to determine the outcomes of early and late PPV for fundus-obscuring VH.

The review adhered to the guidelines established by the Preferred Reporting Items for Systematic Reviews and Meta-Analyses (PRISMA), which provides a framework for transparent and comprehensive reporting of systematic reviews. Although the review protocol was not initially registered in the study design, a registration number has since been assigned to the review. This means that the study has been officially registered with a relevant authority or database, contributing to heightened transparency and lowered research bias.

To identify relevant articles, a systematic literature search was carried out in June 2023 using controlled vocabulary and specific keywords related to “vitrectomy”, “PPV”, “vitreous surgery”, “vitreoretinal surgery”, “vitreous hemorrhage”, or “vitreous haemorrhage”. The search was conducted in electronic databases such as Ovid Medline, Embase (Ovid), the Cochrane Register of Controlled Trials, and the Cochrane Database of Systematic Reviews.

Additionally, we conducted a manual review of the reference lists of the identified articles to identify any potentially pertinent studies that might not have been found through electronic searches. This process helped to ensure that we comprehensively covered all available literature.

After compiling the electronic list of extracted papers, three reviewers (G.B., G.A., and A.G.) independently evaluated the titles and abstracts, identifying articles that met the inclusion criteria. The software “Rayyan” was used as an automation tool for the selection of papers [13]. These criteria enclosed studies reporting the outcomes of PPV for fundus-obscuring vitreous hemorrhage, with a specific focus on the timing of PPV. Exclusion criteria were pilot studies, review studies, case reports, case series with fewer than 12 patients, photo essays, and studies published in languages other than English. Studies involving cadavers, pediatric populations, or animals were also excluded. In cases of disagreement among the reviewers, consensus was reached, and proficient reviewers (F.C. and A.D.M.) were consulted as necessary to provide additional expertise. No unpublished data were solicited from the corresponding authors of the selected articles; the analysis was conducted exclusively based on available published information.

The Oxford Centre for Evidence-Based Medicine (OCEBM) [14] guidelines from 2011 were followed when evaluating the strength of the evidence. These recommendations offer a methodical framework for evaluating the reliability and validity of evidence in medical research. The Grading of Recommendations Assessment, Development, and Evaluation (GRADE) system [15], a tool created to evaluate the veracity of the evidence and facilitate the formulation of recommendations, was used to assess the quality of the evidence.

In summary, our systematic review rigorously followed the methodologies outlined by the PRISMA guidelines. It engaged a comprehensive search strategy to identify relevant studies, clearly outlined inclusion and exclusion criteria, and utilized established frameworks to assess both the level and quality of evidence.

## 3. Results

Data synthesis was unavailable due to the diversity of the available data and differences in research designs.

### 3.1. PPV for Diabetic VH

Summanen et al. [16] in 1989 conducted a retrospective study on 124 eyes of 105 patients with diabetes treated with PPV for dense VH and/or tractional RD. Eyes within the VH-group that achieved a post-operative VA of ≥0.8 had a shorter pre-operative observation period compared with those achieving the best VA < 0.8 (0.7 ± 0.4 vs. 1.4 ± 0.2 years, *p* < 0.05). They concluded that the time interval from VH to surgery was not related to long-term visual outcome and the only discriminating factor was macular detachment.

Feman et al. [17], in 1990, published the results of a randomized controlled trial (RCT) involving 616 eyes with severe diabetic VH comparing early vitrectomy and late PPV. In the early group, PPV was performed immediately, whereas in the late group, PPV was performed after 12 months from the diagnosis. After a follow-up of 4 years, the percentage of patients with a VA of ≥10/15 or more was 21.9% in the early group and 13.0% in the late group, with a statistically significant difference. No statistical difference was identified for patients with a VA of 10/10 or worse than 10/15. Similarly, no statistical difference between groups emerged regarding post-operative complications. They concluded that early PPV provides an advantage over late PPV in terms of VA.

Fassbender et al. [18], in 2016, conducted a retrospective study involving 46 eyes affected by non-clearing PDR-associated VH; 17 eyes underwent PPV after an average time of 14.8 days, and 29 eyes after an average time of 629.6 days. In both groups, VA improved after 6 and 12 months without any difference between the groups.

Taskintuna et al. [19] in 2020 conducted a retrospective study involving 89 eyes afflicted by diabetic VH. The eyes were categorized according to the treatments they underwent: control group (observation only), intravitreal bevacizumab (IVB) injections, PPV, and preoperative single IVB injection followed by PPV. In the PPV groups, the surgical procedure was performed within one month of the initial presentation. In the preoperative IVB-before-PPV group, IVB injections were administered 48–72 h prior to the PPV surgeries. The proportion of eyes gaining ≥ 2 lines was higher in the IVB-before-PPV group (*p* = 0.005) and in the PPV group (*p* = 0.017) compared with the control.

Antoszyk et al. [20], in 2020, presented an RCT with 205 eyes comparing intravitreal aflibercept (IVA) injections versus PPV for diabetic VH. Eyes assigned to PPV underwent surgery within 2 weeks of randomization, and in eyes receiving IVA, vitrectomy was performed if there was persistent VH causing vision impairment following two monthly injections. VA at 4 weeks was 52.6 ETDRS letters in the aflibercept group and 62.3 ETDRS letters in the PPV group. VA improved faster with PPV, but there was no difference between the two treatments at 24 weeks.

Abd Elhamid et al. [21], in 2020, conducted an RCT with 34 eyes with diabetic VH. The first group of eyes underwent three IVAs before PPV, while the second group underwent immediate PPV. At the conclusion of the 9-month follow-up, no difference in VA was observed; however, there was an increase in the final VA in both groups (*p* < 0.001).

### 3.2. PPV for VH Related to Terson Syndrome

Garweg et al. [22], in 2009, performed a retrospective study of 44 patients treated via PPV for TS. Patients who were operated on within 90 days of VH achieved better final VA than their counterparts with delayed/late vitrectomy (*p* = 0.03), and no difference in complications emerged between the early and late vitrectomy groups.

Narayanan et al. [23], in 2017, proposed a retrospective study with 28 VH associated with TS. Patients in the early group underwent PPV before a mean of 40 days from the diagnosis, while patients in the late group had a median of 133 days. The study reported no difference in VA after a follow-up of 1 and 8 months between the two groups; however, in the late group, one eye developed an epiretinal membrane (3.6% of the entire cohort) and three eyes developed an RD at the time or after the initial PPV (10.7% of the entire cohort). No post-operative complications were noted in the early group.

Liu et al. [24], in 2020, presented a retrospective study with 54 eyes that underwent PPV for VH associated with TS. Early PPV was performed in 32 eyes before 3 months from intracranial hemorrhage, while late vitrectomy was performed after 3 months. In all cases, VA at the final follow-up was significantly improved compared with that at presentation (*p* < 0.005), and there was no difference between the early and late groups. In relation to epiretinal membrane and peripheral retina changes (tears and degenerations), these were more common in the delayed/late group (*p* < 0.05).

Nazarali et al. [8], in 2020, performed a retrospective study in 14 eyes with VH associated with TS. Patients were divided into early (before 90 days) and late PPV (after 90 days). After a 3-month follow-up, the final VA showed no significant difference between the groups.

### 3.3. PPV for VH and Presumptive Retinal Tear

Dhingra et al. [25], in 2007, presented a retrospective study of 16 patients treated with PPV for spontaneous non-traumatic VH with presumptive retinal tear. All patients treated within 28 days from the diagnosis obtained a VA of 6/9 or better.

Tan et al. [3], in 2010, conducted a retrospective study of 39 patients treated with early vitrectomy for VH, presumably associated with retinal tears. PPV took place after a mean delay of 2.7 days after presentation, and VA improved post-operatively to 0.8 (*p* < 0.001). This study showed the critical significance of opting for early vitrectomy given the elevated risk of a concealed retinal tear within a dense VH. Furthermore, early PPV provides good outcomes with acceptable complication rates.

### 3.4. PPV for Non-Diabetic, Non-Traumatic VH

Melamud et al. [10], in 2015, presented an observational comparative study in which 66 eyes underwent PPV for VH presumed due to PVD. PPV was performed within 7 days from presentation in the early vitrectomy group and after 7 days in the delayed/late group. The patients in the former achieved a significantly better VA compared with those in the latter group (*p* < 0.05).

Zhang et al. [6], in 2017, performed a retrospective study based on 105 eyes with non-traumatic and non-diabetic VH. Patients underwent surgery if a retinal tear or RD was noted, or if VA remained low and did not improve over 1–3 weeks. Vitrectomy was able to identify the cause of VH and to improve VA (*p* < 0.001). It was suggested that conservative treatment in cases of prolonged and dense VH can lead to significant visual impairment due to the harmful impact of the bleeding on the retina. As a result, early vitrectomy surgery can effectively address this issue by removing the cloudy vitreous, restoring a clear visual path, and improving vision.

Mason et al. [26], in 2019, conducted a retrospective study with 109 eyes affected by hemorrhagic posterior vitreous detachment. No significant difference in VA was reported regarding the timing of surgery, but there was a statistical difference in the RDs between patients who underwent observation compared with those who underwent early vitrectomy (11.63% versus 1.52%, respectively; *p* < 0.05).

Hayashida et al. [11], in 2019, performed a retrospective study with 88 eyes with VH of unclear etiology; 41 eyes underwent PPV before 2 weeks from diagnosis, while 47 were treated after 2 weeks. This study reported a statistically higher VA after 1 month of follow-up in the early PPV group versus the late group (*p* = 0.020), but no statistical difference was found in the post-operative complications among the two groups.

Foo et al. [27], in 2022, presented a retrospective study with 96 eyes comparing observation versus early PPV versus late PPV for non-diabetic VH. In the early group, vitrectomy was performed within a mean of 19.81 days, while in the late group it was performed within a mean of 126 days. Only the early vitrectomy group showed a statistically significant increase in VA after PPV, and no significant difference emerged between groups regarding VA after 12 months of follow-up. There were no significant differences in terms of post-operative complications between the groups either.

### 3.5. PPV for VH Related to Eales Disease

Kumar et al. [28], in 2000, proposed an RCT with 40 cases of VH comparing early vitrectomy and deferred vitrectomy for VH. PPV was performed after 3 to 6 months in the early group and after 6 months in the deferred group. After a follow-up of at least 3 months, the percentage of patients with VA ≥ 6/9 was 65% in the early group and 20% in the deferred group (*p* < 0.001).

**Figure 1:**
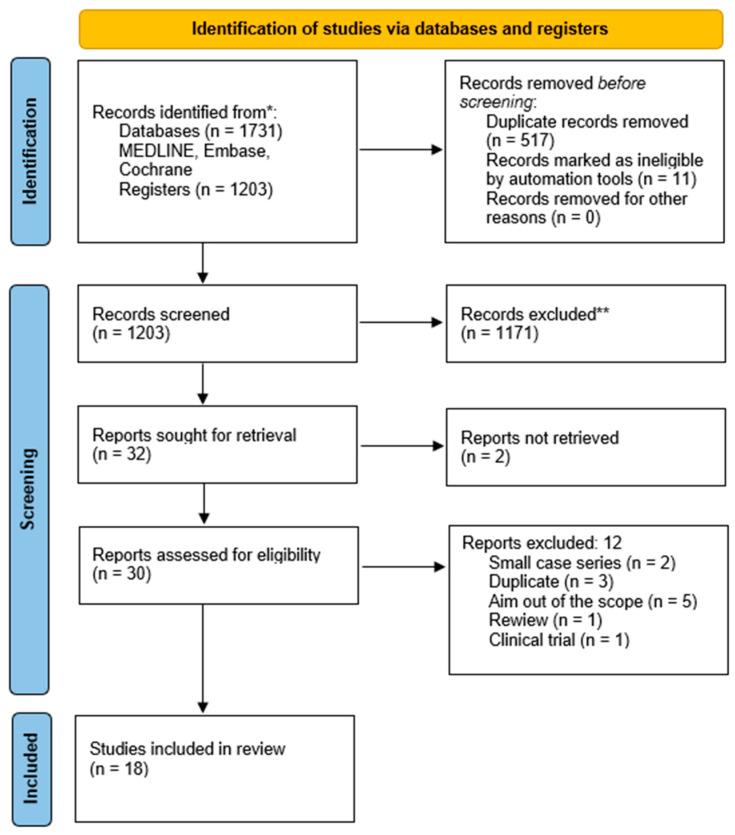
Flowchart of the literature search and selection according to Preferred Reporting Items for Systematic Reviews and Meta-Analyses guidelines (PRISMA). * Consider, if feasible to do so, reporting the number of records identified from each database or register searched (rather than the total number across all databases/registers). ** If automation tools were used, indicate how many records were excluded by a human and how many were excluded by automation tools.

Figure 1 summarizes the research approach applied in this systematic review within a flowchart.

The results of the analysis are collected and displayed in Table 1. Supplementary Material file provides a summary of the reasons for selecting each article.

## 4. Discussion

A step towards better management of patients with VH can be attained by a clear understanding of the potential complications arising from the timing of PPV administration [30]. The findings of this systematic review strengthen the importance and the advantages of early vitrectomy. Prompt surgical intervention appears to lead to better visual outcomes, likely due to the elimination of blood-induced opacities within the vitreous, which obstruct the visual pathway [31]. There is a reduced incidence of post-operative complications in the early vitrectomy group, while secondary complications such as tractional RD and neovascular glaucoma are associated with prolonged VH [32].

The benefits of early PPV in VH are supported by the landmark study by Thompson et al. [31], where they compared outcomes of early and deferred vitrectomy in patients with diabetes having VH. They found that early vitrectomy resulted in significantly better visual outcomes and a lower incidence of complications compared with deferred vitrectomy [33].

Furthermore, early PPV contributes to a lower likelihood of rebleeding events, as the source of hemorrhage is directly addressed and the underlying pathology, such as PDR, is managed simultaneously [34]. This contrasts with late vitrectomy cases, where delayed intervention may allow the progression of retinal pathologies and inflict an increased risk of recurrent bleeding. All the included studies demonstrate that early PPV is at least non-inferior than late or deferred PPV and that such intervention significantly reduces the incidence of recurrence. This emphasizes the importance of early intervention in addressing the essential cause of VH and preventing its recurrence.

Fassbender et al. [18] provided evidence that vitrectomy for diabetic VH can be carried out even earlier than previously recommended, reducing the period during which patients experience impaired vision, thus minimizing the necessity for recurrent office visits, and potentially lowering the requirement for additional in-office procedures. While many retina surgeons now opt to do surgery within a month from VH, this study presents evidence that an early intervention is both safe and efficient.

Connors et al. [35] compared the surgical and visual results for patients who had an early versus delayed/late vitrectomy for dense, non-diabetic, non-vascular VH of unknown origin. Early vitrectomy was described as surgical intervention occurring within 10 days of diagnosis, and late vitrectomy was described as occurring after 10 days. They concluded that the danger of RD was considerable in phakic individuals with extensive bleeding, which should warrant considering a more rapid surgical intervention. In their study, however, multiple etiologies of VH were excluded, whereas we have considered them here.

Patient satisfaction scores further support the superiority of early vitrectomy, as this group experienced quicker visual recovery and a more rapid return to daily activities [36]. The psychological impact of improved vision and independence cannot be understated, and this is an essential consideration in the overall management of VH. A study by Okamoto et al. [37] highlighted that patients who underwent early vitrectomy reported higher levels of satisfaction and a better quality of life compared with those who underwent late vitrectomy.

Furthermore, it is worth considering the significance of recent advancements in vitrectomy equipment, which have significantly improved the surgical approach, making the PPV procedure safer and highly successful [38]. In particular, modern vitrectomy machines are characterized by improved cut rates, allowing for finer control over the cutting and suctioning forces, and enabling surgeons to perform delicate procedures with greater precision and safety. Additionally, the use of smaller gauge instruments makes PPV less invasive. This not only reduces the risk of infection, but also leads to faster post-operative recovery times, and less discomfort for the patient [39]. Moreover, advanced vitrectomy systems often include high-definition imaging and illumination probes, providing surgeons with better visualization of the retina and vitreous, while allowing for more accurate and safer procedures [38]. Despite the growing body of evidence in favor of early PPV, it is important to acknowledge the potential limitations of this approach. Not all cases of VH may require immediate surgical intervention, and careful patient selection is important. Additionally, early PPV may carry risks, including intraoperative bleeding, RD, and endophthalmitis. The use of intravitreal anti-VEGFs specifically for the purpose of reducing intraoperative bleeding during surgery is an area of ongoing research and investigation. Clinical studies exploring this concept and its potential benefits have been on the rise in recent years, and several surgeons are adopting this strategy to mitigate risks associated with vitrectomy [40]. Specifically, numerous studies have been conducted in patients with PDR and VH, demonstrating a reduction in intraoperative bleeding and yielding better outcomes [30]. However, the exact protocols, dosages, and timing of anti-VEGF administration for this purpose have not been firmly established. The decision to pursue early PPV should be based on a thorough assessment of each patient’s clinical presentation, underlying pathologies, and potential benefits. It is also important to highlight that in retrospective studies of early versus late vitrectomy, the late vitrectomy group had already excluded the eyes in which the hemorrhage resolved spontaneously during follow-up. Comparing the early and late vitrectomy groups retrospectively might therefore present a bias. By performing an early vitrectomy, the surgeon may overtreat these patients who may have spontaneous healing of the hemorrhage.

Furthermore, fundus-obscuring VH may be secondary to a plethora of diseases and etiologies and summarizing that can result in biases.

In the literature, there is no clear time definition of when PPV in VH should be defined as “early” or “late”. Vitrectomy has been defined as late very differently, some authors assuming late being >3 months, while others >6 or 12 months after the event. In our opinion, this question is important and remains open for future research to establish a clear temporal definition.

## 5. Conclusions

Our systematic review provides compelling evidence in favor of early PPV for VH secondary to a variety of etiologies. The benefits of improved VA, reduced post-operative complications, lower rebleeding rates, and enhanced patient satisfaction make a strong case for advocating early surgical intervention. While further prospective studies are warranted to corroborate our findings, the current evidence supports a possible shift toward considering early vitrectomy as the preferred approach in the management of VH in selected cases, in particular, cases secondary to conditions that can progress into a more complex clinical picture, such as secondary VH due to diabetes, where the progression to tractional RD is possible, or in retinal tears capable of evolving into an RD. This approach holds the potential to significantly improve visual outcomes and overall quality of life for patients affected by this sight-threatening condition.

## Figures and Tables

**Table 1:** Characteristics, quality, and level of evidence of the included studies.

Author (et al.)	Year	Study Design	Study Sample (Eyes)	Type of Surgery	Outcome Final BCVA	Mean Days from Presentation to Surgery (Patients Were Divided According to the Mean Weeks from Presentation to Surgery; <1 Week: +, between 1–2 Weeks: ++, from 2–12 Weeks: +++, from 12–24 Weeks: ++++, after 24 Weeks/6 Months +++++)	Complications	GRADE [29]
Summanen et al. [16]	1989	Retrospective, comparative, non-randomized	124	PPV for VH versus PPV for central TRD	Eyes within the VH-group that achieved a post-operative visual VA of 0.8 or higher had a shorter pre-operative observation period compared with those achieving the best VA below 0.8 (0.7 ± 0.4 vs. 1.4 ± 0.2 years, *p* < 0.05).	+++++	N/A	Low
Feman et al. [17]	1990	Multicenter, randomized clinical trial	616	Early versus deferred vitrectomy for VH	At 4 years, VA of 10/15 or better in 21.89% of the early vitrectomy and in 13.00% of the deferred vitrectomy. The difference is statistically significant. No difference for VA = 10/10 or VA < 10/15.	Early group: + Deferred group: +++++	At 4 years, for the early and deferred groups, respectively: 17% and 14% of enucleation, phthisis, retrobulbar alcohol injection; 1% and 0% of sympathetic uveitis or endophthalmitis; 15% and 10% of corneal edema, epithelial abnormality; 25% and 16% of neovascular glaucoma; and 16% and 29% of retinal detachment. No statistical difference between groups.	High
Kumar et al. [28]	2000	Randomized clinical trial	40	Early vitrectomy versus deferred vitrectomy for VH	VA ≥ 6/9 in 65% in the early group, and 20% in the deferred group (*p* < 0.001).	Early group: ++++ Deferred group: +++++	Posterior sub-capsular cataract in 15% of the patients in the deferred group, none in the early group; macular pucker in 10% of the early group, 40% in the deferred group; macular edema in 10% of the early group, and 35% in the deferred group.	Moderate
Dhingra et al. [25]	2007	Retrospective case series	16	PPV for spontaneous non-traumatic dense VH	VA ≥ 6/9 in 100% of the early group (*p* < 0.001).	Early group: + Deferred group: ++++	At a mean follow-up of 9 months, two patients needed to repeat vitrectomy: one for an entry site hole and one for a new break. One patient needed cataract surgery.	Very low
Garweg et al. [22]	2009	Retrospective, observational, case series	44	Vitrectomy for Terson syndrome	Patients who were operated on within 90 days of VH achieved better final VA compared with those who underwent delayed vitrectomy (*p* = 0.03): BCVAs were 0.87 ± 0.27 and 0.66 ± 0.31, respectively.	++++	At a follow-up of 23.2 ± 26.5 months, 9% had to undergo reoperations because of proliferative vitreoretinopathy-associated RD. No difference in complications between groups	Very low
Tan et al. [3]	2010	Retrospective, observational, case series	40	Vitrectomy for VH	The median VA improved post-operatively to 0.8 (*p* < 0.001).	+	At a mean follow-up of 23.2 ± 26.5, 9 out of 30 phakic eyes developed cataracts; one patient had to be reoperated because of a macular pucker; two eyes developed an RRD	Very low
Melamud et al. [10]	2015	Retrospective, observational, comparative, case series	66 underwent vitrectomy; 92 eyes were included	Early vitrectomy (30 eyes) versus delayed vitrectomy (36 eyes)	Patients who underwent early vitrectomy achieved significantly better VA compared with those in the delayed group (*p* < 0.05).	Early group: + Deferred group: ++	NA	Low
Fassbender et al. [18]	2016	Retrospective, observational, case series	46	Immediate vitrectomy (17 eyes) versus delayed vitrectomy (29 eyes) for non-clearing, PDR-associated VH	VA improved at the 6-month or 12-month follow-up time in both groups but there was no difference between groups.	Early group: ++ Deferred group: +++++	VH (five eyes), epiretinal membrane (one eye), ocular hypertension that subsequently resolved (one eye), and secondary glaucoma (one eye) occurred within a year. Three out of twelve (25%) of the phakic eyes underwent cataract surgery within a year.	Low
Narayanan et al. [23]	2017	Retrospective, observational case series	28	Early vitrectomy (8 eyes) versus late vitrectomy (20 eyes) for VH associated with TS	No difference in VA at the 1- and 8-month follow-ups between groups.	Early group: +++ Deferred group: +++++	One eye in the postponed group developed an epiretinal membrane (3.6% of the overall cohort), and three eyes (10.7% of the entire cohort) experienced retinal detachments at the time of or shortly after the initial vitrectomy.	Very low
Zhang et al. [6]	2017	Retrospective, observational case series	105	Vitrectomy for VH with non-traumatic and non-diabetic retinopathy	During the mean follow-up period of 14.5 months, the median VA improved post-operatively and was 0.22 logMAR (*p* < 0.001).	Early group: + Deferred group: ++	During the mean follow-up period of 14.5 months, 20 of the 70 phakic eyes developed cataracts. Elevated IOP was present in 10 eyes, among which one with CRVO developed neovascular glaucoma. Seven eyes required reintervention, three of which were for a macular pucker and four because of an inferior rhegmatogenous retinal detachment. Additionally, recurrences of vitreous hemorrhage were observed one month after surgery in two eyes: one because of polypoidal choroidal vasculopathy and one for a retinal arterial macroaneurysm.	Low
Mason et al. [26]	2019	Retrospective, observational case series	109	Early vitrectomy (66 eyes) versus observation (43 eyes) in patients with HPVD	The median VA in the early vitrectomy group was 20/25 and the median VA in the deferred group was 20/20. No significant difference between groups.	N/A	The median follow-up time was 45.5 days for the early vitrectomy group and 65 days for the observation group. RDs occurred more frequently in the observation group (11.63%) compared with the early group (1.52%) (*p* < 0.05).	Low
Hayashida et al. [11]	2019	Retrospective, observational case series	88	Early (41 eyes) versus delayed (47 eyes) vitrectomy for VH	Median VA at 1 month after PPV was 0.21 ± 0.43 logMAR in the early group and 0.66 ± 0.97 logMAR in the delayed group (*p* = 0.020).	Early group: ++ Deferred group: +++	At the 1-month checkup, two eyes in the early group, four eyes in the delayed group, and four eyes in the endophthalmitis group all developed retinal tears or RD; two eyes in the early group, four eyes in the delayed group, and one eye in the delayed group. There were no differences between the groups in terms of complications.	Low
Liu et al. [24]	2020	Retrospective, observational case series	54	Early (32 eyes) versus delayed (22 eyes) vitrectomy for VH associated with TS	In all cases, VA at the final follow-up was significantly improved compared with that at presentation (*p* < 0.005), but there was no difference between the early and late groups.	Early group: +++ Deferred group: ++++	The delayed group had a higher prevalence of epiretinal membrane and peripheral retina changes (tears and degenerations) than the early group (*p* < 0.05). There were no discernible differences between the early and late vitrectomy groups in terms of PVD, RD, retinal exudation, tamponade, and the frequency of post-operative complications.	Low
Nazarali et al. [8]	2020	Retrospective chart review	14	Early vitrectomy (6 eyes) versus late vitrectomy (6 eyes) for Terson syndrome	logMAR of 0.43 ± 0.78 in the early group and 0.61 ± 0.88 in the late group. Final VA did not significantly differ between groups at three months FU.	Early group: +++ Deferred group: ++++	After surgery, posterior vitreous detachment with a membrane at the macula occurred in one patient of the late group. Cataracts developed in four eyes (two in each group).	Very low
Taskintuna et al. [19]	2020	Retrospective cohort study	89	Observation (23 eyes) versus intravitreal bevacizumab injections (29 eyes) versus PPV (17 eyes) versus preoperative single IVB injection before PPV (20 eyes) for diabetic VH	The proportion of eyes gaining ≥ 2 lines was higher than the control in the IVB-before-PPV group (*p* = 0.005) and in the PPV group (*p* = 0.017).	PPV group: +++ In the preoperative IVB-before-PPV group, eyes received IVB injections 48–72 h before the PPV surgeries	During the follow-up period, recurrent hemorrhage occurred in one eye (4.3%) from the control group and in eleven eyes (37.9%) from the IVB group. In both the PPV and preoperative IVB-before-PPV groups, eight eyes (47.1%) and seven eyes (35%), respectively, experienced post-operative VH. Furthermore, one eye from each of the PPV and preoperative IVB-before-PPV groups underwent a secondary PPV procedure. Three eyes in the PPV group and five eyes in the preoperative IVB-before-PPV group required IVB injections for post-operative VH. Two eyes in the PPV group underwent cataract surgery during the follow-up period.	Low
Antoszyk et al. [20]	2020	Randomized, multicenter prospective, clinical trial	205	IVA injections (100 eyes) versus PPV with PRP (105 eyes) for diabetic VH	VA at 4 weeks was 52.6 ETDRS letters in the aflibercept group and 62.3 ETDRS letters in the PPV group. VA improved faster with vitrectomy but there was no difference at 24 weeks.	PPV group: ++ Aflibercetp group: ++++ (PPV was performed if there was persistent vitreous hemorrhage)	Recurrent vitreous hemorrhage occurred in 16 eyes (15%) in the vitrectomy group and 48 eyes (49%) in the aflibercept group at the 2-year follow-up. In the aflibercept group, endophthalmitis affected one eye (1%) while it affected two eyes (2%) in the PPV group. In the aflibercept group, four eyes (4%) had new or worsened rhegmatogenous retinal detachment, whereas in the PPV group, five eyes (5%) did. In the aflibercept group, 23 eyes (31%) underwent cataract extraction, and in the PPV group, 22 eyes (27%) underwent cataract extraction. A total of 42% of participants in the aflibercept group and 41% of participants in the vitrectomy group reported having at least one serious systemic adverse event.	Moderate
Abd Elhamid et al. [21]	2020	Randomized, prospective, clinical trial	34	Three IVA injections followed by PRP (group I, 17 eyes) versus PPV (group II, 17 eyes) for diabetic VH	No difference in VA was found at the end of the follow-up (9 months), but VA improved in both groups (*p* < 0.001).	Group I: +++ Group II: +	At the 9-month follow-up point, there was a statistically significant difference in the recurrence rate between group I (29.4%) and group II (11.8%) (*p* < 0.05 for both). Two eyes in group I (11.8%) and two eyes in group II (11.8%) were found to have an epiretinal membrane. Cataracts developed in four out of eleven phakic eyes (36.4%) in group II. In group II, two eyes developed intraoperative retinal tears (11.8%) and one had significant intraoperative bleeding (5.9%). An amount of 20% SF6 gas was utilized in three eyes (17.6%) in group II.	Moderate
Foo et al. [27]	2022	Retrospective observational single-center cohort study	96	Observation (19 eyes) versus early PPV (72 eyes) versus late PPV (5 eyes) for NDVH	The difference between initial and final VAs was significant only in the early PPV group (*p* = 0.00001), whereas it was not significant in both the conservative group and the late PPV group (respectively, *p* = 0.066 and *p* = 0.46). The difference between groups in the final VA was not significant.	Early group: +++ Late group: ++++	At 12 months follow-up, two eyes developed RRD and one re-bleeding in the early group, two eyes developed retinal tears, one retinal detachment, one neovascular glaucoma, and two cases of persistent vitreous hemorrhage were reported in the conservative group. No statistical difference between groups.	Low

## Data Availability

Data are available upon reasonable request by the corresponding authors.

## Supplementary Materials

The following supporting information can be downloaded at: https://www.mdpi.com/article/10.3390/jcm12206652/s1, please see Supplementary Material file.
