# Supplementary material for: Early versus Late Pars Plana Vitrectomy in Vitreous Hemorrhage: A Systematic Review
Source: J Clin Med. 2023 Oct 20;12(20):6652. doi: 10.3390/jcm12206652 (PMC10607253; doi:10.3390/jcm12206652)
Supplement: Supplementary file 1 [file jcm-12-06652-s001.zip › jcm-2590746-supplementary.pdf]

Documentation of literature search

Research question: Does early vitrectomy in vitreous hemorrhage lead to a better prognosis than late vitrectomy?

The following databases were searched:

**Database**	**Number of Retrieved References**
MEDLINE (Ovid):	534
Embase (Ovid):	947
Cochrane Central Register of Controlled Trials:	236
ClinicalTrials.gov:	14
Number of references before deduplication:	1731
Number of references after deduplication:	1214

All searches were carried out on 6 June 2023 by Toril M. Hestnes, senior librarian, University of Oslo, Library of Medicine and Science

Search syntax:

**Ovid-Databases**	
exp/	Exploded index term.
/	After an index term indicates a subject heading was selected.
.ti,ab,kf.	Search for a term in the title, abstract, and author keywords.
.kw.	=keyword heading.
*	At the end of a term indicates that this term has been truncated, diet* retrieves both diet, diets, dietary.
adj3	Search for two terms next to each other, in any order, up to 3 words in between.
**Cochrane Library**	
ti,ab,kw	Search for a word in the title, abstract, or keyword.
NEAR/3	Search for two terms next to each other, in any order, up to three words in between.

ClinicalTrials.gov:

vitrectom* OR PPV OR "vitreous surgery" OR "vitreoretinal surgery" OR "vitreous hemorrhage" OR "vitreous haemorrhage"

Ovid MEDLINE(R) ALL 1946 to 5 June 2023

#	Searches	Results
1	exp Vitreous Hemorrhage/	2093
2	((vitreous or corpus vitreum or preretinal) adj2 (hemorrhage* or haemorrhage*)).ti,ab,kf.	4413
3	1 or 2	5159
4	exp Vitrectomy/	15818
5	(vitrectom* or phacovitrectom*).ti,ab,kf.	18847
6	PPV.ti,ab,kf.	23760
7	or/4–6	45202
8	3 and 7 [Vitreous hemorrhage AND Vitrectomy. Search could be stopped here]	2196
9	exp Time-to-Treatment/	9807
10	((time or timing or delay*) adj2 (((vitreous or vitreoretinal) adj2 (surg* or intervention*)) or vitrectom* or phacovitrectom* or PPV*)).ti,ab,kf.	378
11	TTT.ti,ab,kf.	1696
12	exp Time Factors/	1229837
13	(time adj (factor* or series)).ti,ab,kf.	49657
14	exp Early Medical Intervention/	3442
15	Disease Progression/	189304
16	((time or after or following) adj2 (onset or presentat* or occurrence*)).ti,ab,kf.	106425
17	((within or after or post* or following or before or advance or prior) adj5 ("3 months" or "three months" or "12 weeks" or "twelve weeks")).ti,ab,kf.	162868
18	((early or earlier or mid-early) adj2 (vs* or versus or compar* or better or superior* or worse or inferior*) adj2 (delay* or late or later or mid-late or post*)).ti,ab,kf.	7209
19	((late or later or mid-late or delay*) adj5 (vitrectom* or PPV* or phacovitrectom* or ((vitreous or vitreoretinal) adj2 (surg* or intervention*)))).ti,ab,kf.	352
20	((early or earlier or mid-early) adj5 (vitrectom* or PPV* or phacovitrectom* or ((vitreous or vitreoretinal) adj2 (surg* or intervention*)))).ti,ab,kf.	599
21	or/9–20	1692935
22	8 and 21	371
23	exp Comparative Study/	1912636
24	(compar* adj (study or studies or analy*)).ti,ab,kf.	204229
25	exp Follow-Up Studies/	691713
26	((follow up or followup) adj7 (study or studies or design or analysis or analyses)).ti,ab,kf.	169029
27	Treatment Outcome/	1148668
28	((visual or clinical or anatomic or surgical or functional) adj2 outcome*).ti,ab,kf.	354380
29	exp Prognosis/	1912878
30	prognos*.ti,ab,kf.	798288
31	exp Retrospective Studies/	1121610
32	(retrospective adj7 (study or studies or design or analysis or analyses or data or review)).ti,ab,kf.	676882
33	exp cohort studies/	2487176
34	(cohort adj (study or studies or analy*)).ti,ab,kf.	329157
35	exp Observational Study/	142432
36	(observational adj (study or studies or analy*)).ti,ab,kf.	164238
37	exp Longitudinal Studies/	165280

38	((longitudinal or long-term or (long adj term)) adj7 (study or studies or design or analysis or analyses or data)).ti,ab,kf.	344640
39	exp Prospective Studies/	660111
40	(prospective adj7 (study or studies or design* or analy* or data)).ti,ab,kf.	548361
41	exp Cross-sectional studies/	468059
42	(cross adj sectional adj7 (study or studies or design or research or analysis or analyses or survey or findings)).ti,ab,kf.	433437
43	exp case-control study/	1420022
44	((case adj control) or (case adj comparison) or (case adj controlled)).ti,ab,kf.	158848
45	(case-referent adj3 (study or studies or design or analysis or analyses)).ti,ab,kf.	637
46	(population adj3 (study or studies or analysis or analyses)).ti,ab,kf.	232304
47	or/23–46	6954244
48	(time or timing or TTT or delay* or early or earlier or mid-early or late or later or mid-late or ((within or after) adj3 (week or weeks or day or days or month or months))).ti,ab,kf.	7210103
49	8 and 47 and (18 or 19 or 20 or 48)	687
50	22 or 49	820
51	exp animals/or exp animal experimentation/or exp animal experiment/or exp models animal/or nonhuman/or exp vertebrate/or exp vertebrates/or exp Cadaver/	26415310
52	exp humans/or exp human experimentation/or exp human experiment/	21285878
53	51 not 52	5130060
54	(veterinary or animal or animals or cadaver* or rabbit or rabbits or rodent or rodents or rat or rats or mouse or mice or rabbit or rabbits or pig or pigs or porcine or pigeon* or horse* or equine or cow or cows or cattle or calves or bovine or goat or goats or donkey* or sheep or ovine or dog or dogs or canine or feline or dolphin* or whale or whales or beetle* or fish or fishes or zebrafish* or bluefish* or seabass).ti.	2457428
55	(human or humans or patient or patients or infant*).ti,ab,kf.	11001362
56	54 not 55	2079266
57	limit 50 to “all children (0 to 18 years)”	195
58	limit 50 to “systematic review”	4
59	limit 50 to “reviews (maximizes specificity)”	10
60	50 not (53 or 56 or 57 or 58 or 59)	613
61	limit 60 to English language	534

Embase Classic+Embase 1947 to 5 June 2023

#	Searches	Results
1	exp vitreous hemorrhage/	8455
2	((vitreous or corpus vitreum or preretinal) adj2 (hemorrhage* or haemorrhage*)).ti,ab,kf.	6319
3	1 or 2	10320
4	exp vitrectomy/or exp pars plana vitrectomy/	30458
5	(vitrectom* or phacovitrectom* or PPV).ti,ab,kf.	65899
6	4 or 5	75503
7	3 and 6 [Vitreous hemorrhage AND Vitrectomy. Search could be stopped here]	4224
8	time to treatment/	25898

9	((time or timing or delay*) adj2 (((vitreous or vitreoretinal) adj2 (surg* or intervention*)) or vitrectom* or phacovitrectom* or PPV*)).ti,ab,kf.	524
10	TTT.ti,ab,kf.	2655
11	time factor/	46290
12	(time adj (factor* or series)).ti,ab,kf.	56321
13	early intervention/	33396
14	disease exacerbation/	181799
15	((time or after or following) adj2 (onset or presentat* or occurrence* or symptom*)).ti,ab,kf.	243509
16	((within or after or post* or following or before or advance or prior) adj5 ("3 months" or "three months" or "12 weeks" or "twelve weeks")).ti,ab,kf.	273134
17	((early or earlier or mid-early) adj2 (vs* or versus or compar* or better or superior* or worse or inferior*) adj2 (delay* or late or later or mid-late or post*)).ti,ab,kf.	11677
18	((late or later or mid-late or delay*) adj5 (vitrectom* or PPV* or phacovitrectom* or ((vitreous or vitreoretinal) adj2 (surg* or intervention*)))).ti,ab,kf.	497
19	((early or earlier or mid-early) adj5 (vitrectom* or PPV* or phacovitrectom* or ((vitreous or vitreoretinal) adj2 (surg* or intervention*)))).ti,ab,kf.	862
20	or/8–19	850554
21	7 and 20	539
22	exp comparative study/	1702999
23	(compar* adj (study or studies or analy*)).ti,ab,kf.	269701
24	follow up/	2099867
25	((follow up or followup) adj7 (study or studies or design or analysis or analyses)).ti,ab,kf.	278722
26	treatment outcome/	955692
27	((visual or clinical or anatomic or surgical or functional) adj2 outcome*).ti,ab,kf.	561868
28	retrospective study/	1481289
29	(retrospective adj7 (study or studies or design or analysis or analyses or data or review)).ti,ab,kf.	1158446
30	cohort analysis/	1042897
31	(cohort adj (study or studies or analy*)).ti,ab,kf.	497728
32	observational study/	330568
33	(observational adj (study or studies or analy*)).ti,ab,kf.	263270
34	longitudinal study/	195316
35	((longitudinal or long-term or (long adj term)) adj7 (study or studies or design or analysis or analyses or data)).ti,ab,kf.	501905
36	prospective study/	882991
37	(prospective adj7 (study or studies or design* or analy* or data)).ti,ab,kf.	852951
38	prognosis/	727999
39	prognos*.ti.	291649
40	cross-sectional study/	566578
41	(cross adj sectional adj7 (study or studies or design or research or analysis or analyses or survey or findings)).ti,ab,kf.	578465
42	case-control study/	206831
43	((case adj control) or (case adj comparison) or (case adj controlled)).ti,ab,kf.	215387
44	(case-referent adj3 (study or studies or design or analysis or analyses)).ti,ab,kf.	705
45	(population adj3 (study or studies or analysis or analyses)).ti,ab,kf.	356733
46	or/22–45	8704374

47	(time or timing or TTT or delay* or early or earlier or mid-early or late or later or mid-late or ((within or after) adj3 (week or weeks or day or days or month or months))).ti,ab,kf.	10282282
48	7 and 46 and (17 or 18 or 19 or 47)	1289
49	21 or 48	1486
50	limit 49 to conference abstract status	175
51	exp animals/or exp animal experimentation/or exp animal experiment/or exp models animal/or nonhuman/or exp vertebrate/or exp vertebrates/or exp Cadaver/	34976281
52	exp humans/or exp human experimentation/or exp human experiment/	26903127
53	51 not 52	8074615
54	(veterinary or animal or animals or cadaver* or rabbit or rabbits or rodent or rodents or rat or rats or mouse or mice or rabbit or rabbits or pig or pigs or porcine or pigeon* or horse* or equine or cow or cows or cattle or calves or bovine or goat or goats or donkey* or sheep or ovine or dog or dogs or canine or feline or dolphin* or whale or whales or beetle* or fish or fishes or zebrafish* or bluefish* or seabass).ti.	3009129
55	(human or humans or patient or patients or infant*).ti.	5064459
56	54 not 55	2889607
57	limit 49 to child	121
58	limit 49 to “systematic review”	20
59	limit 49 to “reviews (maximizes specificity)”	16
60	49 not (50 or 53 or 56 or 57 or 58 or 59)	1174
61	limit 60 to English language	947

Cochrane Central Register of Controlled Trials (Wiley):

#1	MeSH descriptor: [Vitreous Hemorrhage] explode all trees	164
#2	((vitreous or corpus vitreum or preretinal) near/2 (hemorrhage* or haemorrhage*)):ti,ab,kw (Word variations have been searched)	581
#3	#1 OR #2	581
#4	MeSH descriptor: [Vitrectomy] explode all trees	714
#5	vitrectom* or phacovitrectom* or PPV	3735
#6	#4 OR #5	3735
#7	#3 AND #6	292
#8	MeSH descriptor: [Time-to-Treatment] explode all trees	561
#9	MeSH descriptor: [Time Factors] explode all trees	73007
#10	MeSH descriptor: [Early Medical Intervention] explode all trees	492
#11	(time or timing or TTT* or delay* or early or earlier or mid-early or late or later or mid-late or week or weeks or day or days or month or months):ti,ab,kw (Word variations have been searched)	1267373
#12	{OR #8-#11}	1267373
#13	#7 AND #12	255

Language: English.

Summary of the 32 records screened for inclusion/exclusion and the determining reasons behind each choice.

	Title	Include (N. of Eyes)	Exclude	Explanation for Exclusion (or Comments)
1	Early vitrectomy for severe vitreous hemorrhage in diabetic retinopathy. Two-year results of a randomized trial. Diabetic Retinopathy Vitrectomy Study report 2. The Diabetic Retinopathy Vitrectomy Study Research Group. Arch Ophthalmol. 1985 Nov;103(11):1644-52. PMID: 2865943.		Excluded	Duplicate
2	Early vitrectomy for severe proliferative diabetic retinopathy in eyes with useful vision. Clinical application of results of a randomized trial—Diabetic Retinopathy Vitrectomy Study Report 4. The Diabetic Retinopathy Vitrectomy Study Research Group. Ophthalmology. 1988 Oct;95(10):1321-34. doi: 10.1016/s0161-6420(88)33014-9. PMID: 2465518.		Excluded	Duplicate
3	Summanen P. Significance of various systemic and ocular parameters in the long-term prognosis after diabetic vitrectomy. Int Ophthalmol. 1989 Sep;13(5):311-9. doi: 10.1007/BF02279867. PMID: 2625379.	One included (124 eyes)		
4	Early vitrectomy for severe vitreous hemorrhage in diabetic retinopathy. Four-year results of a randomized trial: Diabetic Retinopathy Vitrectomy Study Report 5. Arch Ophthalmol. 1990 Jul;108(7):958-64. doi: 10.1001/archopht.1990.01070090060040. Erratum in: Arch Ophthalmol 1990 Oct;108(10):1452. PMID: 2196036.	Two included (616 eyes)		
5	Flynn HW Jr, Chew EY, Simons BD, Barton FB, Remaley NA, Ferris FL 3rd. Pars plana vitrectomy in the Early Treatment Diabetic Retinopathy Study. ETDRS report number 17. The Early Treatment Diabetic Retinopathy Study Research Group. Ophthalmology. 1992 Sep;99(9):1351-7. doi: 10.1016/s0161-6420(92)31779-8. PMID: 1407968.		Excluded	Duplicate
6	Verbraeken H, Van Egmond J. Non-diabetic and non-oculotraumatic vitreous haemorrhage treated by pars plana vitrectomy. Bull Soc Belge Ophtalmol. 1999;272:83-9. PMID: 10427924.		Excluded	Aim out of the scope: early vitrectomy was performed only for cases with a retinal detachment or a suspected retinal tear
7	Kumar A, Tiwari HK, Singh RP, Verma L, Prasad N. Comparative evaluation of early vs. deferred vitrectomy in Eales' disease. Acta Ophthalmol Scand. 2000 Feb;78(1):77-8. doi: 10.1034/j.1600-0420.2000.078001077.x. PMID: 10726795.	Three included (40 eyes)		
8	Results of the Early Treatment Diabetic Retinopathy Study (ETDRS) Follow-up Study Chew EY, Ferris FL, Csaky KG, Murphy RP, Schachat A, Bigio M, Thompson D IOVS, 2000, 41, ARVO Abstract 5056		Excluded	Not retrievable

9	Makiuchi, R, and Uemura, A, and Doi, N, and Nakao, K. (2001). Long-term outcome of early vitrectomy for juvenile proliferative diabetic retinopathy. 55. 163-168.	Excluded	Only 9 cases
10	Ness T, Janknecht P, Berghorn C. Frequency of ocular hemorrhages in patients with subarachnoidal hemorrhage. Graefes Arch Clin Exp Ophthalmol. 2005 Sep;243(9):859-62. doi: 10.1007/s00417-005-1131-z. Epub 2005 Mar 15. PMID: 16158309.	Excluded	Aim out of the scope: vitreous hemorrhage secondary to subarachnoidal hemorrhage, no differentiation according to timing of surgery
11	Dhingra N, Pearce I, Wong D. Early vitrectomy for fundus-obscuring dense vitreous haemorrhage from presumptive retinal tears. Graefes Arch Clin Exp Ophthalmol. 2007 Feb;245(2):301-4. doi: 10.1007/s00417-006-0278-6. PMID: 16802133.	Four included (16 eyes)	
12	Garweg JG, Koerner F. Outcome indicators for vitrectomy in Terson syndrome. Acta Ophthalmol. 2009 Mar;87(2):222-6. doi: 10.1111/j.1755-3768.2008.01200.x. Epub 2008 Jun 3. PMID: 18537934.	Five included (45 eyes)	
13	Tan HS, Mura M, Bijl HM. Early vitrectomy for vitreous hemorrhage associated with retinal tears. Am J Ophthalmol. 2010 Oct;150(4):529-33. doi: 10.1016/j.ajo.2010.04.005. Epub 2010 Jun 25. PMID: 20579632.	Six included (40 eyes)	
14	Zenoni S, Comi N, Fontana P. Individualised treatment of proliferative diabetic retinopathy: optimal surgical timing improves long-term outcomes. EPMA J. 2010 Mar;1(1):78-81. doi: 10.1007/s13167-010-0007-4. Epub 2010 Mar 10. PMID: 23199043; PMCID: PMC3405311.	Excluded	Review
15	Ratnarajan G, Mellington F, Saldanha M, de Silva SR, Benjamin L. Long-term visual and retinopathy outcomes in a predominately type 2 diabetic patient population undergoing early vitrectomy and endolaser for severe vitreous haemorrhage. Eye (Lond). 2011 Jun;25(6):704-8; quiz 709. doi: 10.1038/eye.2011.65. Epub 2011 Apr 15. PMID: 21494282; PMCID: PMC3171789.	Excluded	Aim out of the scope: Vitrectomy for VH in patients with diabetes type 1 versus in patients with diabetes type 2
16	Melamud A, Pham H, Stoumbos Z. Early Vitrectomy for Spontaneous, Fundus-Obscuring Vitreous Hemorrhage. Am J Ophthalmol. 2015 Nov;160(5):1073-1077.e1. doi: 10.1016/j.ajo.2015.07.025. Epub 2015 Jul 21. PMID: 26209230.	Seven included (92 eyes)	
17	Fassbender JM, Ozkok A, Canter H, Schaal S. A Comparison of Immediate and Delayed Vitrectomy for the Management of Vitreous Hemorrhage due to Proliferative Diabetic Retinopathy. Ophthalmic Surg Lasers Imaging Retina. 2016 Jan;47(1):35-41. doi: 10.3928/23258160-20151214-05. PMID: 26731207.	Eight included (134 eyes)	

18	Narayanan R, Taylor SC, Nayaka A, Deshpande R, St Aubin D, Hrisomalos FN, Hu J, Rajagopal R, Tewari A, Apte RS. Visual Outcomes after Vitrectomy for Terson Syndrome Secondary to Traumatic Brain Injury. Ophthalmology. 2017 Jan;124(1):118-122. doi: 10.1016/j.ophtha.2016.09.009. Epub 2016 Nov 3. PMID: 27817917.	Nine included (28 eyes)		
19	Zhang T, Zhang J, Sun X, Tian J, Shi W, Yuan G. Early vitrectomy for dense vitreous hemorrhage in adults with non-traumatic and non-diabetic retinopathy. J Int Med Res. 2017 Dec;45(6):2065-2071. doi: 10.1177/0300060517708942. Epub 2017 Jun 19. PMID: 28627981; PMCID: PMC5805203.	Ten included (105 eyes)		
20	Mason LB, Wilhite JB, McGwin G Jr, Swain TA, Crosson JN. Comparison of Observation Versus Vitrectomy for Patients With Hemorrhagic Posterior Vitreous Detachment. Ophthalmic Surg Lasers Imaging Retina. 2019 Nov 1;50(11):e288-e293. doi: 10.3928/23258160-20191031-16. PMID: 31755980.	Eleven included (109 eyes)		
21	Hayashida M, Miki A, Imai H, Otsuka K, Azumi A, Nakamura M. Impact of Early Vitrectomy for Dense Vitreous Hemorrhage of Unknown Etiology. Ophthalmologica. 2019;242(4):234-238. doi: 10.1159/000501723. Epub 2019 Sep 3. PMID: 31480058.	Twelve included (88 eyes)		
22	Liu X, Yang L, Cai W, Gao L, Li Y. Clinical features and visual prognostic indicators after vitrectomy for Terson syndrome. Eye (Lond). 2020 Apr;34(4):650-656. doi: 10.1038/s41433-019-0547-3. Epub 2019 Aug 27. PMID: 31455901; PMCID: PMC7093525.	Thirteen included (54 eyes)		
23	Petrarca R, Soare C, Wong R, Desai R, Neffendorf J, Simpson A, Jackson TL. Intravitreal ranibizumab for persistent diabetic vitreous haemorrhage: a randomised, double-masked, placebo-controlled feasibility study. Acta Ophthalmol. 2020 Dec;98(8):e960-e967. doi: 10.1111/aos.14282. Epub 2019 Nov 1. PMID: 31674134.		Excluded	Aim out of the scope: aflicercept injection vs. placebo injection before vitrectomy
24	Nazarali S, Kherani I, Hurley B, Williams G, Fielden M, Adatia F, Kherani A. OUTCOMES OF VITRECTOMY IN TERSON SYNDROME: A Multicenter Canadian Perspective. Retina. 2020 Jul;40(7):1325-1330. doi: 10.1097/IAE.0000000000002570. PMID: 31145391.	Fourteen included (14 eyes)		
25	Schreur V, Brouwers J, Van Huet RAC, Smeets S, Phan M, Hoyng CB, de Jong EK, Klevering BJ. Long-term outcomes of vitrectomy for proliferative diabetic retinopathy. Acta Ophthalmol. 2021 Feb;99(1):83-89. doi: 10.1111/aos.14482. Epub 2020 Jul 9. PMID: 32643273; PMCID: PMC7891313.		Excluded	Aim out of the scope: to evaluate long-term outcomes of vitrectomy
26	Diabetic vitrectomy is safer and is applicable at an earlier stage of the disease https://dx.doi.org/10.1586/eop.12.21		Excluded	Not retrievable
27	Taskintuna I, Elsayed MEAA, Taskintuna K, Ahmad K, Khandekar R, Schatz P, Kozak I. Comparison of	Fifteen		

	outcomes of four different treatment modalities for diabetic vitreous haemorrhage. Sci Rep. 2020 Feb 28;10(1):3674. doi: 10.1038/s41598-020-60378-8. PMID: 32111892; PMCID: PMC7048724.	included (89 eyes)		
28	Antoszyk AN, Glassman AR, Beaulieu WT, Jampol LM, Jhaveri CD, Punjabi OS, Salehi-Had H, Wells JA 3rd, Maguire MG, Stockdale CR, Martin DF, Sun JK; DRCR Retina Network. Effect of Intravitreous Aflibercept vs. Vitrectomy With Panretinal Photocoagulation on Visual Acuity in Patients With Vitreous Hemorrhage From Proliferative Diabetic Retinopathy: A Randomized Clinical Trial. JAMA. 2020 Dec 15;324(23):2383-2395. doi: 10.1001/jama.2020.23027. PMID: 33320223; PMCID: PMC7739132.	Sixteen included (205 eyes)		
29	Abd Elhamid AH, Mohamed AAEA, Khattab AM. Intravitreal Aflibercept injection with Panretinal photocoagulation versus early Vitrectomy for diabetic vitreous hemorrhage: a randomized clinical trial. BMC Ophthalmol. 2020 Apr 6;20(1):130. doi: 10.1186/s12886-020-01401-4. PMID: 32252674; PMCID: PMC7137269.	Seventeen included (34 eyes)		
30	Foo E, Grassi P, Spiteri-Cornish K. Early vitrectomy in eyes with non-diabetic vitreous hemorrhage. Ther Adv Ophthalmol. 2022 Apr 29;14:25158414221090099. doi: 10.1177/25158414221090099. PMID: 35510165; PMCID: PMC9058341.	Eighteen included (96 eyes)		
31	Severe Proliferative Diabetic Retinopathy NCT01115257 https://clinicaltrials.gov/study/NCT01115257		Excluded	Clinical trial, no result posted
32	Makiuchi, R, and Uemura, A, and Doi, N, and Nakao, K (2001). Long-term outcome of early vitrectomy for juvenile proliferative diabetic retinopathy. 55. 163-168.		Excluded	Only 9 cases
